# C1GALT1, Negatively Regulated by miR-181d-5p, Promotes Tumor Progression via Upregulating RAC1 in Lung Adenocarcinoma

**DOI:** 10.3389/fcell.2021.707970

**Published:** 2021-07-07

**Authors:** Xiaoxia Dong, Yongyu Liu, Xinzhou Deng, Jun Shao, Shuangyue Tian, Shuang Chen, Rongxin Huang, Ziao Lin, Chunli Chen, Li Shen

**Affiliations:** ^1^Department of Clinical Oncology, Taihe Hospital, Hubei University of Medicine, Shiyan, China; ^2^Institute of Basic Medical Sciences, Hubei University of Medicine, Shiyan, China

**Keywords:** lung cancer, C1GALT1, miR-181d-5p, RAC1, growth, metastasis

## Abstract

Glycosyltransferases are frequently dysregulated in lung cancer. Core 1 β 1, 3-galactosyltransferase 1 (C1GALT1), an enzyme highly expressed in various cancers, is correlated with tumor initiation and development. However, the role of C1GALT1 in lung cancer remains poorly understood. In this study, through bioinformatic analysis and clinical validation, we first discovered that C1GALT1 expression was upregulated in lung adenocarcinoma (LUAD) tissues and was closely related to poor prognosis in patients with LUAD. Gain- and loss-of-function experiments showed that C1GALT1 promoted LUAD cell proliferation, migration, and invasion *in vitro*, as well as tumor formation *in vivo*. Further investigation demonstrated that RAC1 expression was positively regulated by C1GALT1 in LUAD, whereas silencing Rac1 could reverse C1GALT1-induced tumor growth and metastasis. Moreover, miR-181d-5p was identified as a negative regulator for C1GALT1 in LUAD. As expected, the inhibitory effects of miR-181d-5p on LUAD cell proliferation, migration, and invasion were counteracted by restoration of C1GALT1. In summary, our results highlight the importance of the miR-181d-5p/C1GALT1/RAC1 regulatory axis during LUAD progression. Thus, C1GALT1 may serve as a potential therapeutic target for LUAD.

## Introduction

According to the global cancer statistics in 2018, lung cancer remains the most frequent cancer and the leading cause of cancer death worldwide (Bray et al., 2018). Based on pathological types, lung cancer can be divided into small cell lung cancer (SCLC, 15% of the cases) and non-small cell lung cancer (NSCLC, 85% of the cases). NSCLC is further classified into three types, namely, lung squamous cell carcinoma (LUSC), lung adenocarcinoma (LUAD), and large cell carcinoma. Lung cancer is a highly aggressive disease and has a poor prognosis, with a 5-year survival rate of less than 20% (Siegel et al., 2020). Although therapeutic strategies (surgery, chemotherapy, radiotherapy, and immunotherapy) have been applied for the treatment of lung cancer, some patients still develop postoperative recurrence and metastasis. Frequent recurrence or metastasis remains a major obstacle for lung cancer treatment (Wang et al., 2019; Zhan et al., 2019). Thus, it is essential to clarify the mechanism underlying the pathogenesis and progression of lung cancer, which may help to improve therapeutic outcomes.

All living cells typically decorate their surfaces with diverse glycans linked to proteins or lipids (Jaiman and Thattai, 2020). These glycans participate in numerous biological events, such as cellular communication, protein folding, and immune regulation (Narimatsu et al., 2019). Cells in different tissues and developmental stages usually express distinct glycans on their surfaces. Unlike RNA transcription and protein translation, glycans are not directly synthesized from a genome-encoded template (Moremen and Haltiwanger, 2019). Glycan biosynthesis is mainly controlled by a series of glycosyltransferases (GTs). GTs are the key enzymes that catalyze the formation of complex glycans by adding one sugar to specific acceptors at a time (Park et al., 2018). Until now, over 200 GTs have been identified in the human genome (Moremen et al., 2018). GTs are frequently altered in various diseases, including cancer (Gloster, 2014; Wu et al., 2019). Remarkably, aberrant expression or dysregulation of GTs also occurs in lung cancer (Reticker-Flynn and Bhatia, 2015; Park et al., 2020). Therefore, elucidating the relationship between GTs and lung cancer is particularly important.

Core 1 β1, 3-galactosyltransferase 1 (C1GALT1), an exclusive T-synthase in mammalian cells, catalyzes the transfer of galactose (Gal) from UDP-Gal to the extant GalNAc forming core 1 O-glycans (Galβ1, 3GalNAcα-O-Ser/Thr) (Liu et al., 2020). Studies have shown that C1GALT1 is abnormally expressed in a variety of malignant tumors, such as gastric cancer (Lee et al., 2020), head and neck cancer (Lin et al., 2018), pancreatic ductal adenocarcinoma (Kuo et al., 2021), laryngeal carcinoma (Dong et al., 2018b), ovarian cancer (Chou et al., 2017), and hepatocellular carcinoma (Wu et al., 2013). However, the role of C1GALT1 in lung cancer remains unclear.

In this study, we comprehensively assessed the biological function of C1GALT1 in lung cancer using bioinformatics tools and clinical samples. We found that C1GALT1 was overexpressed in LUAD tissues and high C1GALT1 expression was associated with poor prognosis in LUAD patients. Through a series of functional experiments *in vitro* and *in vivo*, we confirmed that C1GALT1 could facilitate LUAD progression. Further mechanistic studies demonstrated that C1GALT1 expression was negatively regulated by miR-181d-5p in LUAD and the promotive effects of C1GALT1 on LUAD progression were mediated by Rac Family Small GTPase 1 (RAC1). These findings may provide novel therapeutic targets for the treatment of lung cancer.

## Materials and Methods

### Clinical Specimens and Cell Lines

Sixty paired LUAD tissues and adjacent normal tissues were collected from patients who underwent surgical resection at Taihe Hospital, Hubei University of Medicine. Samples were immediately frozen in liquid nitrogen and stored at –80°C. This study was approved by the Ethics Committee of the Hubei University of Medicine. Informed consent was obtained from all subjects.

LUAD cell lines (A549, H1299, H1975, and H441) and a normal lung epithelial cell line (BEAS-2B) were purchased from the Procell (Wuhan, China). All cells were cultured in DMEM (Gbico, Detroit, MI, United States) supplemented with 10% fetal bovine serum (Hyclone, Logan, UT, United States). Cells were maintained at 37°C, 5% CO_2_.

### Bioinformatics Analysis

The miRNA and mRNA expression datasets (level 3) were downloaded from The Cancer Genome Atlas (TCGA^1^). Data visualization was performed using the ggplot2 R package. Survival analysis was conducted using the “survminer” and “survival” R packages. Correlation matrices were constructed using the R package “corrplot.” ROC curves were plotted using the “pROC” package. The prognostic nomogram was generated by the “rms” package. Venn diagram was made using the VennDiagram R package. Gene set enrichment analysis (GSEA) was performed with the GSEA software^2^. *p* < 0.05 was considered statistically significant and *p* > 0.05 was considered no significant.

### Cell Transfection

The C1GALT1-overexpressed plasmid (OV) or empty pcDNA3.1 plasmid (Mock), lentiviral vectors expressing C1GALT1 short hairpin RNAs (shRNAs) or negative control shRNA (shNC), RAC1 small interfering RNA (siRNA) or non-silencing siRNA (siNC), miR-181d-5p inhibitor, inhibitor negative control (inhibitor NC), miR-181d-5p mimic and mimic negative control (mimic NC) were purchased from GenePharma (Shanghai, China). Cell transfection was conducted using Lipofectamine 3000 (Invitrogen, Carlsbad, CA, United States) or RNAi-mate reagent (GenePharma), following the manufacturer’s guidelines. Stably transfected cells were selected by G418 or puromycin. Sequences of shRNAs and siRNAs are listed in Supplementary Table 1.

### Quantitative Real-Time PCR (qPCR)

Total RNA was isolated from clinical samples or cell lines using RNAiso Plus (Takara, Kusatsu, Japan), according to the manufacturer’s instructions. For quantification of mRNA, Reverse transcription and PCR amplification was performed using PrimeScript RT Master Mix (Takara) and SYBR Green PCR Master Mix (Takara). For miRNA analysis, TaqMan miRNA Reverse Transcription Kit (Applied Biosystems, Foster City, CA, United States) and TaqMan miRNA Assay Kit (Applied Biosystems) were used. Data were normalized to GAPDH or U6 using the 2^−ΔΔCt^ method. Primers for qPCR are listed in Supplementary Table 2.

### Western Blot

Total protein was extracted using RIPA lysis buffer (Beyotime, Shanghai, China). After quantification, an equal amount of protein was run on a 10% SDS-PAGE gel and transferred to a PVDF membrane (Millipore, Billerica, MA, United States). After blocking with 5% BSA, the membrane was probed with the indicated antibodies. Primary antibodies used in this study were as follows: C1GALT1 (Abcam, Cambridge, United Kingdom, ab237734), RAC1 (ab155938), and GADPH (ab9485). Reactive bands were visualized using an ECL system (Pierce, Rockford, IL, United States). RAC1 activity was determined using the Rac1 activation assay kit (Cytoskeleton, Denver, CO, United States). GTP-RAC1 was detected by Western blot using an anti-RAC1 antibody.

### Immunohistochemistry (IHC)

Tumor tissues were embedded in paraffin using standard techniques. IHC was carried out as previously described (Dong et al., 2018a, b). The IHC score (range 0–12) was calculated by multiplying the intensity and the percentage of staining. A total score > 5 was defined as high expression, and ≤ 4 was regarded as low expression. Images were captured using an Olympus BX53 microscope (Olympus, Tokyo, Japan).

### Proliferation Analysis

Cell Counting Kit-8 (CCK-8, Dojindo, Japan) and colony formation assays were applied for determining cell proliferation. For the CCK-8 assay, cells were seeded into 96-well plates at a density of 3 × 10^3^ per well. Absorbance at 450 nm was recorded using a microplate reader (Bio-Rad, Hercules, CA, United States). For colony formation assay, cells (1 × 10^3^ per well) were plated in 6-well plates. After 2 weeks, colonies (>50 cells) were fixed with methanol and stained with 1% crystal violet (Beyotime).

### Migration and Invasion Analysis

Cell migratory and invasive abilities were determined using uncoated or Matrigel-coated Transwell chambers (Corning Inc., Corning, NY, United States), respectively. Protocols were identical to those described previously (Dong et al., 2018b). After 24 h of incubation, the migrated and invaded cells in lower chambers were counted under a light microscope (magnification, 100×). The wound-healing assay was also done to assess cell migration. Artificial wounds were created by scraping using a sterile 200 μl pipette tip. The wound areas were photographed at 0 and 24 h.

### Cell Cycle and Apoptosis Analysis

Cell Cycle Detection Kit (KeyGene, Nanjing, China) was utilized to monitor the cell cycle distribution. Cells were stained with PI staining solution containing RNase. Annexin V-PE/7AAD Apoptosis Detection Kit (BD Biosciences, Franklin Lakes, NJ, United States) was used to analyze apoptotic cells (Annexin-V + and 7AAD-). Data were acquired on a FACScan flow cytometer (BD Biosciences) and analyzed using FlowJo software (Treestar, Ashland, OR, United States).

### Luciferase Assay

The pmirGLO vectors with wild-type or the mutant miR-181d-5p binding site in C1GALT1 3’UTR were designed and constructed by GenePharma. Subsequently, miR-181d-5p mimic or NC mimic were co-transfected with the above reporter vectors into cells using Lipofectamine 3000. After 48 h of transfection, the luciferase activity was detected using the Dual-Luciferase Reporter Assay System (Promega, Madison, WI, United States).

### Mice Xenograft Models

Female BALB/c nude mice (3–4 weeks) were obtained from the Animal Center of Hubei University of Medicine. Stably transfected cells (5 × 10^6^) were subcutaneously injected into nude mice. Tumor volumes were evaluated every week. Tumor size was calculated according to the formula tumor volume = (length × width^2^)/2. All mice were sacrificed after 4 weeks. All animal experiments were approved by the Institutional Animal Care and Use Committee of the Hubei University of Medicine.

### Statistical Analysis

Data were analyzed using GraphPad Prism 7.0 (GraphPad Software, La Jolla, CA, United States) and presented as mean ± SD from at least three independent experiments. Statistical significances were determined using the Student’s *t*-test, one-way analysis of variance (ANOVA), Pearson correlation analysis, or Kaplan-Meier survival analysis. *p* < 0.05 was considered statistically significant.

## Results

### C1GALT1 Is Overexpressed in LUAD and Predicts Poor Prognosis

**FIGURE 1:**
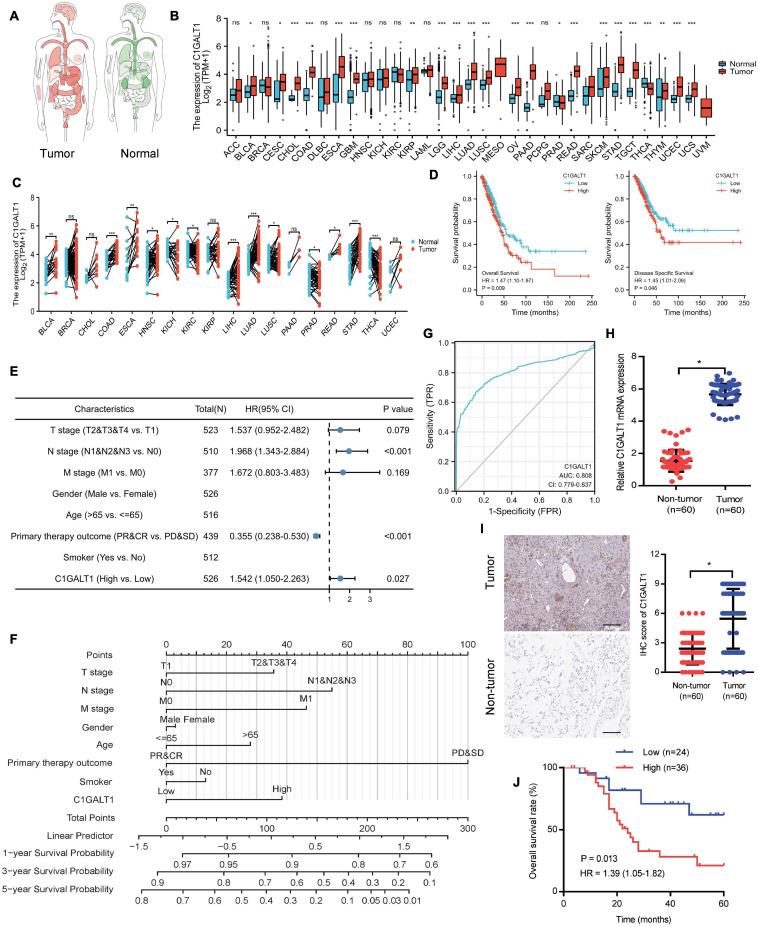
C1GALT1 is overexpressed in LUAD and associated with poor prognosis. **(A)** Pan-cancer analysis of C1GALT1 using the TCGA database. **(B)** C1GALT1 expression in unpaired tumor and normal tissues from the TCGA database. **(C)** C1GALT1 expression profiles across all tumor samples and paired normal tissues in the TCGA database. **(D)** Kaplan-Meier curves for overall survival and disease-specific survival according to C1GALT1 expression in the TCGA-LUAD cohort. **(E)** Forest plot of multivariate Cox regression analysis. **(F)** Nomogram for predicting 1-, 3- and 5-year survival probability of LUAD patients. **(G)** ROC curve of C1GALT1 in LUAD. **(H)** Analysis of C1GALT1 expression in LUAD tissues and adjacent non-tumor tissues by qPCR. **(I)** IHC analysis of C1GALT1 expression in LUAD samples. Scale bar: 100 μm. **(J)** Overall survival analysis of LUAD patients based on differential expression levels of C1GALT1. *p < 0.05; **p < 0.01; ***p < 0.001; *ns* = not significant.

To explore the expression pattern of C1GALT1 in lung cancer, we performed bioinformatics analysis using the TCGA database. The results showed that C1GALT1 expression was higher in the majority of tumors than normal tissues, particularly in the stomach, lung, and esophageal (Figures 1A–C). Notably, C1GALT1 was highly expressed in both LUAD and LUSC tissues, two major subtypes of NSCLC. Kaplan-Meier survival analysis demonstrated that high C1GALT1 expression was significantly negatively correlated with overall survival and disease-specific survival in LUAD patients but not in LUSC patients (Figure 1D and Supplementary Figure 1). Thus, we mainly focused on the role of C1GALT1 in LUAD. Univariate and multivariate Cox regression analysis confirmed that C1GALT1 overexpression was an independent prognostic factor for overall survival in LUAD patients (Figure 1E and Table 1). Using the multivariate Cox proportional hazard models, we successfully constructed a prognostic nomogram to predict 1-, 3-, and 5-year survival probability in LUAD (Figure 1F). We then used ROC curve analysis to assess the diagnostic value of C1GALT1. We found that C1GALT1 was able to distinguish LUAD patients from healthy controls, with the AUC value of 0.808 (Figure 1G). Subsequently, qPCR and IHC were utilized to validate the data obtained from TCGA. We observed that C1GALT1 expression was upregulated in LUAD tissues compared with adjacent non-tumor tissues (Figures 1H,I). LUAD patients with high C1GALT1 expression had shorter overall survival times than those with low C1GALT1 expression (Figure 1J). Moreover, C1GALT1 expression was closely related to lymph node metastasis and TNM stage (Table 2). Collectively, the above findings highlighted the importance of C1GALT1 in LUAD development and progression.

**TABLE 1:** Univariate and multivariate Cox proportional analysis for overall survival.

Characteristics	Total (N)	Univariate analysis		Multivariate analysis	
			
		Hazard ratio (95% CI)	*P*-value	Hazard ratio (95% CI)	*P*-value
T stage (T2&T3&T4 vs. T1)	523	1.728 (1.229–2.431)	0.002	1.537 (0.952–2.482)	0.079
N stage (N1&N2&N3 vs. N0)	510	2.601 (1.944–3.480)	< 0.001	1.968 (1.343–2.884)	< 0.001
M stage (M1 vs. M0)	377	2.136 (1.248–3.653)	0.006	1.672 (0.803–3.483)	0.169
Gender (Male vs. Female)	526	1.070 (0.803–1.426)	0.642		
Age (> 65 vs. < = 65)	516	1.223 (0.916–1.635)	0.172		
Primary therapy outcome (PR&CR vs. PD&SD)	439	0.377 (0.268–0.530)	< 0.001	0.355 (0.238–0.530)	< 0.001
Smoker (Yes vs. No)	512	0.894 (0.592–1.348)	0.591		
C1GALT1 (High vs. Low)	526	1.473 (1.103–1.966)	0.009	1.542 (1.050–2.263)	0.027

**TABLE 2:** Relevance analysis of C1GALT1, RAC1, and miR-181d-5p expression in LUAD patients.

Variables	C1GALT1		*P*-value	RAC1		*P*-value	miR-181d-5p		*P*-value
					
	Low (24)	High (36)		Low (20)	High (40)		Low (37)	High (23)	
**Age**									
<65	10	18	0.167	9	19	0.386	20	8	0.249
≥65	14	18		11	21		17	15	
**Gender**									
Male	11	16	0.305	14	13	0.181	18	9	0.063
Female	13	20		6	27		19	14	
**Tumor size (cm)**									
<5	7	12	0.108	10	9	0.072	14	5	0.059
≥5	17	24		10	31		23	18	
**Smoking status**									
No	12	15	0.225	7	20	0.144	15	12	0.077
Yes	12	21		13	20		22	11	
**Lymph node metastasis**									
N0	19	6	0.023	13	12	0.018	12	13	0.011
N1-3	5	30		7	28		25	10	
**TNM stage**									
I + II	18	13	0.008	12	19	0.005	13	18	0.004
III + IV	6	23		8	21		24	5	

### C1GALT1 Promotes the Growth of LUAD *in vitro*

**FIGURE 2:**
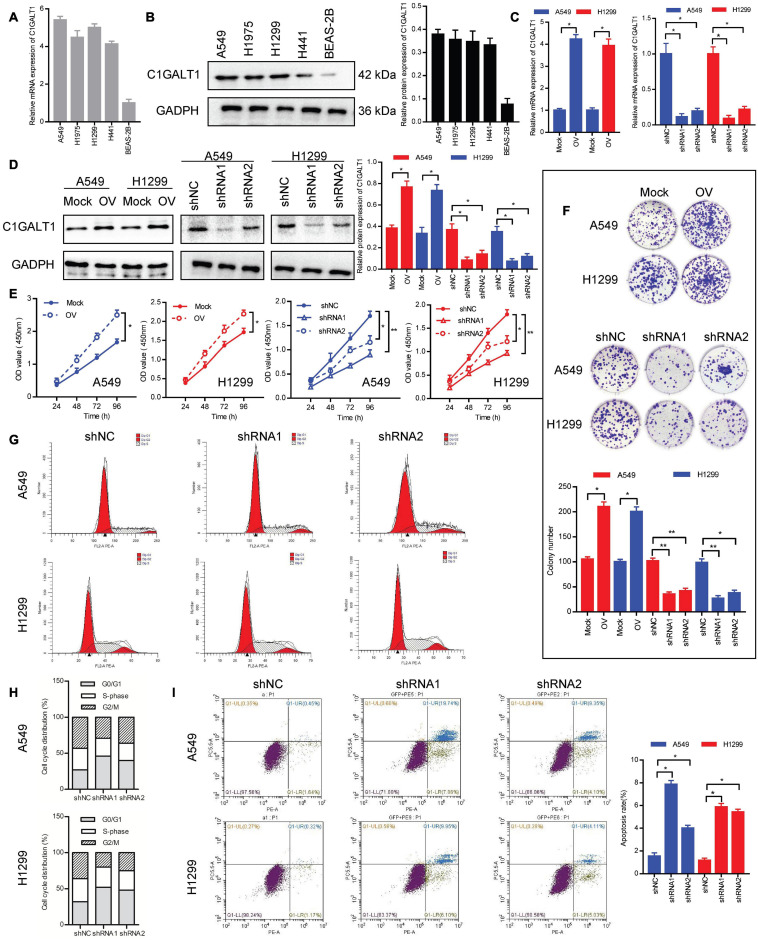
C1GALT1 promotes LUAD cell growth *in vitro.* **(A,B)** Analysis of C1GALT1 expression in four LUAD cell lines and one normal lung cell line by qPCR **(A)** and Western blot **(B)**. **(C,D)** C1GALT1 transfection efficiency was determined by qPCR **(C)** and western blot **(D)**. **(E,F)** Cell proliferation was assessed by CCK-8 assay **(E)** and colony formation assay **(F)**. **(G)** Flow cytometry analysis of cell cycle. **(H)** Quantitative analysis of cell cycle distribution. **(I)** Analysis of apoptosis by flow cytometry. Mock, cells transfected with empty plasmid; OV, cells transfected with C1GALT1 overexpression plasmid; shNC, cells infected with negative control shRNA; shRNAs, cells infected with C1GALT1 shRNA. Data are expressed as mean ± SD. *p < 0.05; **p < 0.001.

To investigate the biological function of C1GALT1 in LUAD, the expression levels of C1GALT1 in four LUAD cell lines (A549, H1299, H1975, and H441) and one normal lung epithelial cell line (BEAS-2B) were examined by qPCR and Western blot. We found that C1GALT1 expression was higher in LUAD cells than in normal lung cells (Figures 2A,B). Considering the cell cultured condition and growth state, A549 and H1299 cells were used in the following experiments. Next, C1GALT1 was stably knocked down or overexpressed in A549 and H1299 cells (Figures 2C,D). CCK-8 assay showed that C1GALT1 overexpression strengthened cell viability, whereas C1GALT1 knockdown weakened cell viability (Figure 2E). Colony formation assay revealed that the number of colonies was increased by C1GALT1 overexpression, and decreased by knockdown of C1GALT1 (Figure 2F). Usually, cell proliferation is regulated by the cell cycle and apoptosis. To determine whether C1GALT1 knockdown-mediated inhibition of cell proliferation was associated with cell cycle arrest or apoptosis induction, the flow cytometry assay was performed. PI staining demonstrated that the cell cycle was arrested at the G0/G1 phase upon C1GALT1 knockdown (Figures 2G,H). Annexin V-PE/7AAD staining showed that knockdown of C1GALT1 could increase the percentage of cells undergoing apoptosis (Figure 2I). These results indicated that C1GALT1 played a critical role in controlling the growth of LUAD cells.

### C1GALT1 Facilitates LUAD Cell Migration and Invasion *in vitro*

**FIGURE 3:**
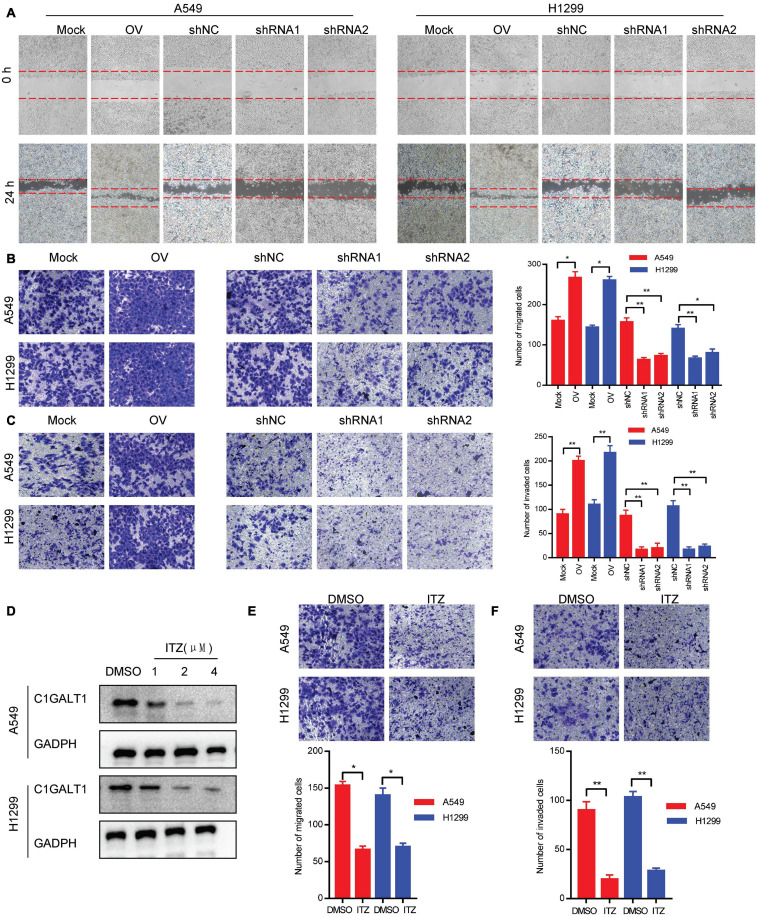
C1GALT1 promotes LUAD cell migration and invasion *in vitro.* **(A,B)** The effect of C1GALT1 on cell migration was measured by wound-healing assay **(A)** and Transwell migration assay **(B)**. **(C)** The effect of C1GALT1 on cell invasion was determined by Matrigel invasion assay. **(D)** Analysis of C1GALT1 expression by Western blot after treatment with itraconazole (ITZ) for 48 h. **(E,F)** The effect of itraconazole (2 μM) on cell migration and invasion was assessed by Transwell migration assay **(E)** and Matrigel invasion assay **(F)**. Mock, cells transfected with empty plasmid; OV, cells transfected with C1GALT1 overexpression plasmid; shNC, cells infected with negative control shRNA; shRNAs, cells infected with C1GALT1 shRNA. Data are expressed as mean ± SD. *p < 0.05; **p < 0.001.

To evaluate the impact of C1GALT1 on LUAD cell metastasis, wound-healing, Transwell migration, and Matrigel invasion assays were carried out. We found that C1GALT1 overexpression accelerated the scratch repair of A549 and H1299 cells, whereas C1GALT1 knockdown had the opposite effects (Figure 3A). Moreover, the migratory and invasive abilities of A549 and H1299 cells were greatly enhanced by overexpression of C1GALT1 and significantly reduced by knockdown of C1GALT1 (Figures 3B,C). It has been reported that itraconazole is a specific inhibitor of C1GALT1 (Lin et al., 2018). We noticed that C1GALT1 protein expression in A549 and H1299 cells was suppressed by itraconazole in a dose-dependent manner (Figure 3D). Additionally, Transwell migration and Matrigel invasion assays showed that itraconazole pretreatment could inhibit the migration and invasion of A549 and H1299 cells (Figures 3E,F). The above-mentioned data suggested that C1GALT1 may function as an oncogene by affecting LUAD cell migration and invasion.

### C1GALT1 Drives LUAD Tumor Growth *in vivo*

**FIGURE 4:**
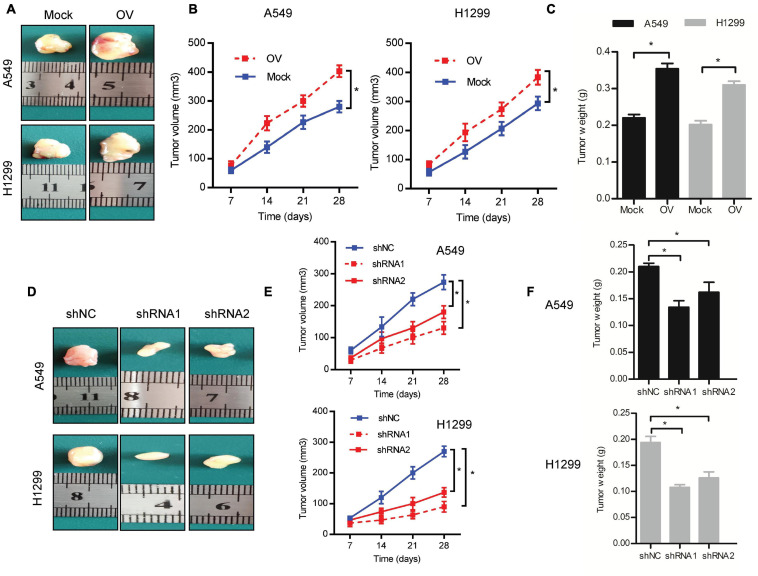
C1GALT1 promotes LUAD tumor growth *in vivo*. **(A)** Representative images of excised tumors from nude mice injected with C1GALT1-overexpressing cells. **(B,C)** Analysis of tumor volume **(B)** and weight **(C)** after C1GALT1 overexpression. **(D)** Representative images of excised tumors from nude mice injected with C1GALT1-knockdown cells. **(E,F)** Analysis of tumor volume **(E)** and weight **(F)** after C1GALT1 knockdown. *n* = 3 mice per group. Mock, cells transfected with empty plasmid; OV, cells transfected with C1GALT1 overexpression plasmid; shNC, cells infected with negative control shRNA; shRNAs, cells infected with C1GALT1 shRNA. Data are expressed as mean ± SD. *p < 0.05.

To determine whether C1GALT1 could affect tumor growth *in vivo*, we conducted a mouse xenograft experiment. The C1GALT1-overexpressing, C1GALT1-knockdown, or respective control cells were subcutaneously injected into the nude mice. All mice were sacrificed on day 28. We found that C1GALT1 overexpression enhanced the tumorigenic abilities of A549 and H1299 cells, as manifested by increased tumor size, volume, and weight (Figures 4A–C). On the contrary, C1GALT1 knockdown in A549 and H1299 cells inhibited the growth of xenograft tumors (Figures 4D–F). The C1GALT1 shRNA1 was selected for subsequent experiments due to its higher efficiency. These *in vivo* findings strengthened and confirmed our *in vitro* results that C1GALT1 contributed to LUAD tumorigenesis.

### C1GALT1 Expression Is Negatively Regulated by miR-181d-5p in LUAD

**FIGURE 5:**
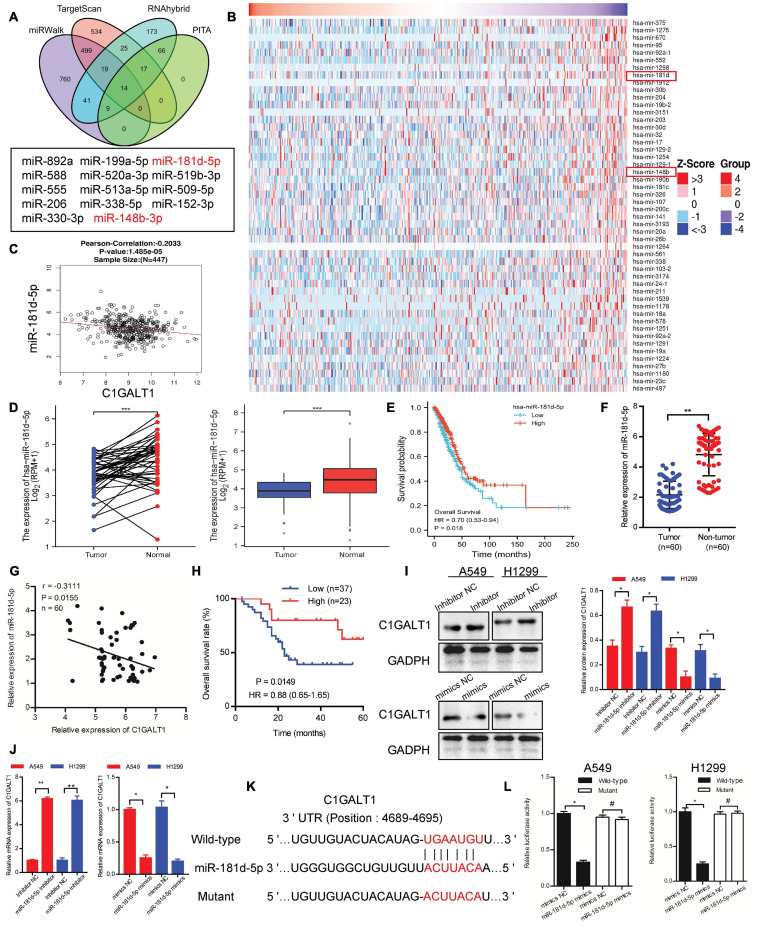
C1GALT1 is directly downregulated by miR-181d-5p in LUAD. **(A)** Venn diagrams showing the number of potential miRNAs targeting the 3’ UTR of C1GALT1. **(B)** Heat maps showing the top 50 miRNAs negatively correlated with C1GALT1 in LUAD. **(C)** Correlation between miR-181d-5p and C1GALT1 in TCGA-LUAD samples. **(D)** miR-181d-5p expression in paired (left) or unpaired (right) LUAD tissues and normal lung tissues from the TCGA database. **(E)** Survival analysis of miR-181d-5p in LUAD from the TCGA database. **(F)** Analysis of miR-181d-5p expression by qPCR in clinical LUAD samples. **(G)** Pearson correlation analysis of miR-181d-5p with C1GALT1 expression in clinical LUAD samples. **(H)** Overall survival analysis of LUAD patients based on miR-181d-5p expression in clinical samples. **(I,J)** Analysis of C1GALT1 expression in LUAD cell lines after transfection with miR-181d-5p mimics or inhibitor by Western blot **(I)** and qPCR **(J)**. **(K)** The potential binding site of miR-181d-5p in the 3’UTR of C1GALT1. **(L)** Relative luciferase activity detection. Data are expressed as mean ± SD.*p < 0.05; **p < 0.01; ***p < 0.001; ^#^*p* > 0.05.

To probe the mechanism behind C1GALT1 upregulation, we first evaluated genomic alterations of this gene through the cBioportal online platform and found that C1GALT1 displayed a very low mutation frequency in LUAD (Supplementary Figure 2). DNA methylation analysis revealed that the C1GALT1 promoter was almost unmethylated in LUAD (Supplementary Figure 3). Hence, we turned our attention to miRNAs, the major regulators of gene expression. Using four publicly available algorithms (PITA, TargetScan, miRWalk, and RNAhybrid), we identified 14 miRNAs that were predicted to target C1GALT1 (Figure 5A). Moreover, we downloaded the miRNA expression profiles of LUAD patients from the TCGA database. The top 50 most significant miRNAs negatively correlated with C1GALT1 in LUAD were visualized by the heat map (Figure 5B). By intersecting the potential target miRNAs and negatively correlated miRNAs, miR-148b-3p, and miR-181d-5p were selected for validation. Although TCGA dataset analysis showed that these two miRNAs were downregulated in LUAD, only the miR-181d-5p expression had a significant correlation with poor prognosis in patients with LUAD (Figures 5C–E and Supplementary Figures 4A–D). LUAD patients with low miR-181d-5p expression had shorter overall survival times than those with high miR-181d-5p expression. These observations were further confirmed by qPCR analysis in our samples (Figures 5F–H and Table 2). Therefore, miR-181d-5p was considered a functional regulator of C1GALT1 in LUAD. We next explored whether C1GALT1 was directly modulated by miR-181d-5p in LUAD cells. The results demonstrated that transfecting miR-181d-5p mimics or inhibitor to A549 and H1299 cell lines could significantly downregulate or upregulate the expression of C1GALT1 in these two cells, respectively (Figures 5I,J and Supplementary Figure 5). Based on the bioinformatics prediction, we identified a putative binding site for miR-181d-5p in the C1GALT1 3’UTR (Figure 5K). As expected, miR-181d-5p mimics inhibited the luciferase activity of wild-type C1GALT1 3’UTR but did not affect the luciferase activity of mutant C1GALT1 3’UTR (Figure 5L). Altogether, these findings indicated that miR-181d-5p was the upstream regulatory factor of C1GALT1, and downregulation of miR-181d-5p resulted in increased C1GALT1 expression in LUAD.

### miR-181d-5p Suppresses the Proliferation, Migration, and Invasion of LUAD Cells by Targeting C1GALT1

**FIGURE 6:**
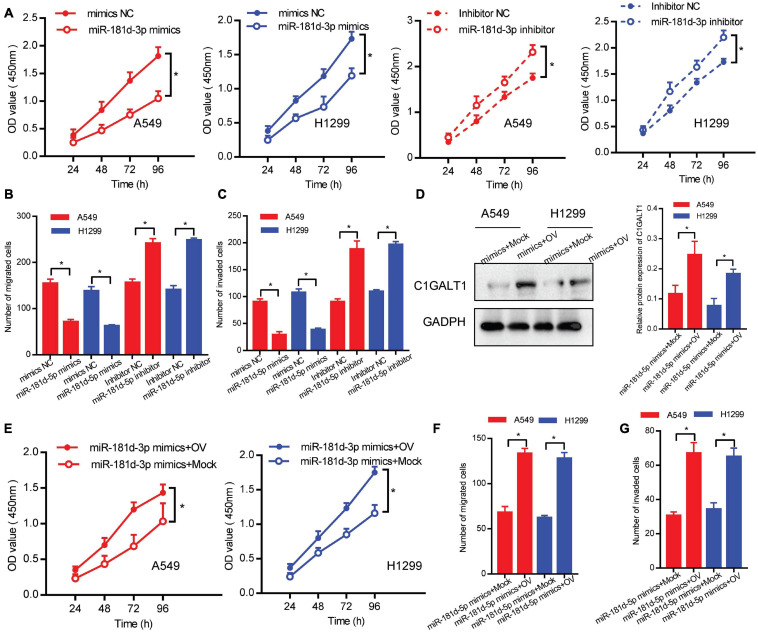
miR-181d-5p functions as a negative regulator of C1GALT1 in LUAD. **(A–C)** The effect of miR-181d-5p mimics or inhibitor on cell proliferation, migration, and invasion was determined by CCK-8 assay **(A)**, Transwell migration assay **(B)**, and Matrigel invasion assay **(C)**. **(D)** Western blot analysis of C1GALT1 expression in cells co-transfected with miR-181d-5p mimics and C1GALT1 overexpression plasmid. **(E–G)** The inhibitory effect of miR-181d-5p mimics on cell proliferation **(E)**, migration **(F)**, and invasion **(G)** were antagonized by overexpression of C1GALT1. Mock, cells transfected with empty plasmid; OV, cells transfected with C1GALT1 overexpression plasmid. Data are expressed as mean ± SD. *p < 0.05.

To assess the role of miR-181d-5p in LUAD, we performed loss- or gain-of-function experiments. Cell proliferation, migration, and invasion abilities were determined by CCK-8, Transwell migration, and Matrigel invasion assays, respectively. We found that miR-181d-5p mimics repressed the proliferation, invasion, and migration of A549 and H1299 cells. Conversely, miR-181d-5p inhibitor promoted LUAD cell proliferation, invasion, and migration (Figures 6A–C). Moreover, A549 and H1299 cells were co-transfected with miR-181d-5p mimics and C1GALT1 overexpression plasmid. The re-expression efficiency of C1GALT1 was verified by Western blot (Figure 6D). Subsequent functional assays showed that the inhibitory effects of miR-181d-5p mimics on cell proliferation, invasion, and migration were attenuated by restoration of C1GALT1 (Figures 6E–G). These results provided further evidence that C1GALT1 was directly regulated by miR-181d-5p in LUAD.

### C1GALT1 Positively Regulates the Expression of RAC1 in LUAD

**FIGURE 7:**
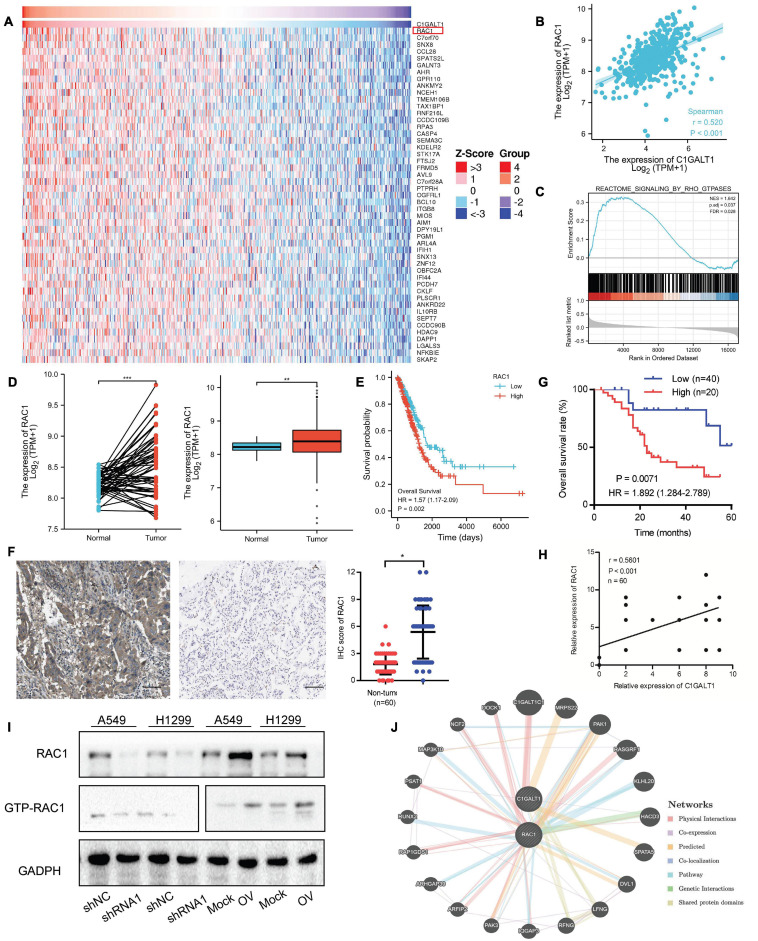
RAC1 expression is positively regulated by C1GALT1 in LUAD. **(A)** Heat maps showing the top 50 genes positively correlated with C1GALT1 in TCGA-LUAD samples. **(B)** Correlation between RAC1 and C1GALT1 in TCGA-LUAD samples. **(C)** GSEA analysis of C1GALT1-associated genes in TCGA-LUAD samples. **(D)** RAC1 expression in paired (left) or unpaired (right) LUAD tissues and normal lung tissues from the TCGA database. **(E)** Survival analysis of RAC1 in LUAD from the TCGA database. **(F)** Analysis of RAC1 expression by IHC in clinical LUAD samples. Scale bar: 100 μm. **(G)** Pearson correlation analysis of RAC1 with C1GALT1 expression in clinical LUAD samples. **(H)** Overall survival analysis of LUAD patients based on RAC1 expression in clinical samples. **(I)** The effect of C1GALT1 on RAC1 expression in LUAD cells was detected by Western blot. **(J)** The network between RAC1 and C1GALT1 was predicted by the online GeneMANIA tool. Mock, cells transfected with empty plasmid; OV, cells transfected with C1GALT1 overexpression plasmid; shNC, cells infected with negative control shRNA; shRNA1, cells infected with C1GALT1 shRNA1. Data are expressed as mean ± SD. *p < 0.05; **p < 0.01; ***p < 0.001.

To understand how C1GALT1 influenced LUAD progression, the genes co-expressed with C1GALT1 in LUAD tissues were analyzed using the TCGA database. The top 50 genes positively and negatively correlated with C1GALT1 were shown in the heat maps (Figure 7A and Supplementary Figure 6). Among these candidates, RAC1 exhibited the highest correlation coefficient (Figure 7B). RAC1 is a member of Rho family small GTPases (Nguyen et al., 2018). GSEA analysis indicated that Rho GTPases signaling was closely associated with C1GALT1 expression (Figure 7C). Meanwhile, RAC1 is a driver of tumor growth and metastasis (De et al., 2019). Therefore, we speculated that C1GALT1 might facilitate LUAD cell proliferation, invasion, and migration by regulating RAC1. To test this hypothesis, we first examined the expression of RAC1 in LUAD through publicly available TCGA data. We found that RAC1 expression in LUAD tissues was significantly higher than that in normal tissues (Figure 7D). We then evaluated the prognostic value of RAC1 in LUAD. We found that high RAC1 expression was associated with poor overall survival (Figure 7E). IHC staining in our samples also confirmed that RAC1 was upregulated in LUAD (Figure 7F and Table 2), and correlated with poor clinical outcome (Figure 7G). Additionally, a significant positive correlation was observed between C1GALT1 and RAC1 expression in clinical samples (Figure 7H). We subsequently investigated whether C1GALT1 could affect RAC1 expression and activation in LUAD cells. We discovered that RAC1 expression and activity in A549 and H1299 cells was augmented by C1GALT1 overexpression and attenuated by C1GALT1 knockdown (Figure 7I and Supplementary Figure 7). Moreover, a gene regulatory network was generated by GeneMANIA to determine the interactive relationship between C1GALT1 and RAC1 (Figure 7J). Taken together, these results suggested that RAC1 was a downstream effector of C1GALT1 in LUAD.

### RAC1 Silencing Reverses C1GALT1-Induced LUAD Growth and Metastasis

**FIGURE 8:**
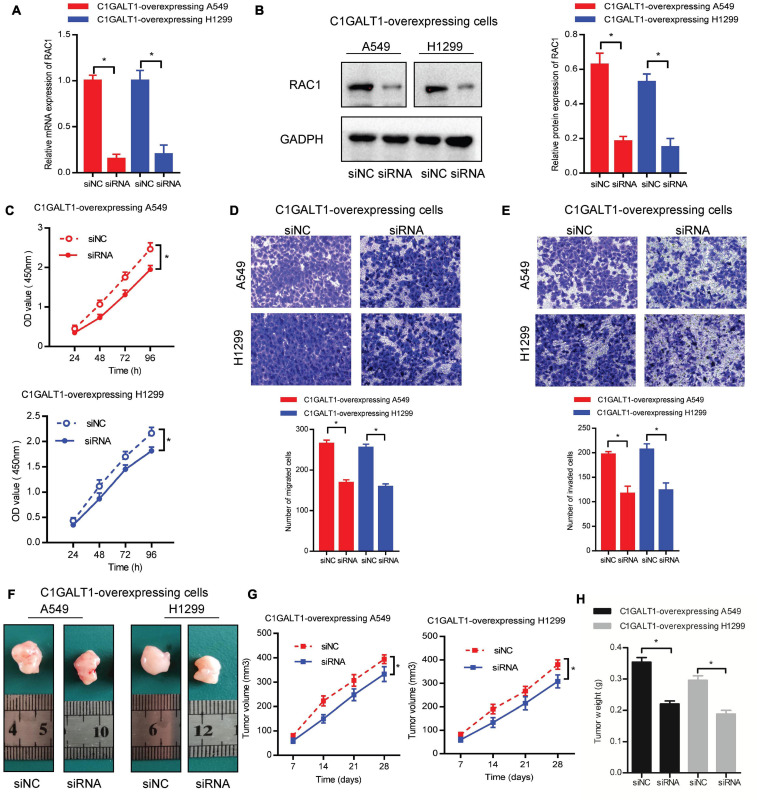
RAC1 serves as a downstream effector of C1GALT1 in LUAD. **(A,B)** Analysis of RAC1 expression in C1GALT1-overexpressing cells after transfection with RAC1 siRNA by qPCR **(A)** and Western blot **(B)**. **(C–E)** The proliferation, migration, and invasion of cells co-transfected with C1GALT1 overexpression plasmid and RAC1 siRNA were determined by CCK-8 assay **(C)**, Transwell migration assay **(D)**, and Matrigel invasion assay **(E)**. **(F)** Representative images of excised tumors from nude mice (*n* = 3 per group). **(G,H)** Tumor volume **(G)** and tumor weight **(H)** were measured. siNC, cells transfected with non-silencing siRNA; siRNA, cells transfected with RAC1 siRNA. Data are expressed as mean ± SD. *p < 0.05.

To confirm the important role of RAC1 in C1GALT1-mediated LUAD progression, we silenced RAC1 with specific siRNA in C1GALT1-overexpressing cells. Silencing efficiency was determined by qPCR and Western blot (Figures 8A,B). Then CCK-8, Transwell migration, and Matrigel invasion assays were used to examine the malignant phenotypes of A549 and H1299 cells. We found that the increased cell proliferation, migration, and invasion induced by C1GALT1 overexpression were weakened by RAC1 silencing (Figures 8C–E). Moreover, the promotion of tumor growth by C1GALT1 overexpression was mitigated by silencing of RAC1 (Figures 8F–H). Our data indicated that RAC1 was a major contributor to the function of C1GALT1 in LUAD growth and metastasis.

## Discussion

Accumulating studies have demonstrated that tumorigenesis and development are closely related to the abnormal expression of GTs (Meech et al., 2019; Akella et al., 2020; Bastian et al., 2021). C1GALT1 is one of the important members of the GT family. Although C1GALT1 is associated with the occurrence, metastasis, and prognosis of various malignancies, the clinical significance, biological function, and regulatory mechanism of C1GALT1 in lung cancer are poorly understood. This study reported for the first time that C1GALT1 was upregulated in LUAD tissues and cell lines. Moreover, C1GALT1 expression was negatively correlated with overall survival and disease-specific survival in patients with LUAD. Our study further confirmed that C1GALT1 could serve as a useful diagnostic and prognostic indicator for LUAD patients. C1GALT1, negatively regulated by miR-181d-5p, was able to promote LUAD progression via upregulating RAC1. Thus, C1GALT1 may play an important role in LUAD progression.

CCK-8, colony formation, wound-healing, Transwell migration, and Matrigel invasion assays were used in experiments involving C1GALT1 overexpression or knockdown. The results showed that the proliferation, migration, and invasion of LUAD cells were enhanced by C1GALT1 overexpression but attenuated by C1GALT1 knockdown. Flow cytometry analysis further revealed that loss of C1GALT1 inhibited the proliferation of LUAD cells via arresting the cell cycle at the G0/G1 phase and inducing apoptosis. In addition, overexpression or knockdown of C1GALT1 could facilitate or suppress tumor growth *in vivo*. It has been reported that C1GALT1 is essential for processes such as cell proliferation, migration, and invasion in a variety of malignancies (Lee et al., 2020; Kuo et al., 2021). Our findings are consistent with previous reports, suggesting a pro-oncogenic role for C1GALT1 in LUAD.

As we all know, miRNAs are a class of small non-coding RNAs that modulate gene expression at the post-transcriptional level (Shin et al., 2020). miRNAs are involved in numerous cellular processes including cell growth, development, differentiation, and apoptosis (Saliminejad et al., 2019; Condrat et al., 2020). Dysregulation of miRNAs in cancer cells has been widely reported (Rupaimoole and Slack, 2017). We, therefore, sought to explore how miRNA affects C1GALT1 expression in LUAD. Using bioinformatic analysis and luciferase activity assays, we identified miR-181d-5p as a negative regulator of C1GALT1. Our gain- and loss-of-function experiments confirmed that miR-181d-5p directly regulated C1GALT1 expression via interaction with its 3’UTR. Meanwhile, a significant negative correlation between miR-181d-5p and C1GALT1 was observed in LUAD tissues. As expected, the inhibitory effects of miR-181d-5p on LUAD cell proliferation, migration, and invasion were counteracted by restoration of C1GALT1. There is some evidence that miR-181d-5p participates in tumorigenesis and malignant transformation via different signaling pathways (Chen et al., 2018; Wang et al., 2020). Of note, miR-181d-5p has been proven to have a tumor-suppressive role in NSCLC (Gao et al., 2019). In this study, we demonstrated that miR-181d-5p acted as a tumor suppressor in LUAD by targeting C1GALT1. To the best of our knowledge, miR-181d-5p was first uncovered to be involved in the regulation of C1GALT1 expression in LUAD.

The endogenous small GTPase, Rac1, is implicated in many cellular activities, such as phagocytosis, adhesion, migration, motility, and proliferation (Zou et al., 2017). Recently, many studies have shown that RAC1 plays a critical role in multiple physiological and pathological processes, including cancer (De et al., 2019). Aberrant expression of RAC1 is recognized as a hallmark of cancer and contributes to the tumorigenic and metastatic phenotypes of cancer cells (Kazanietz and Caloca, 2017). A growing body of evidence suggests that RAC1 can be regulated by various factors. For example, lncRNA NR2F2-AS1 overexpression mediated the upregulation of Rac1 in clear cell renal cell carcinoma (Chen et al., 2020). Knockdown of RhoGDI2 reduced the mRNA expression of Rac1 in gastric cancer (Zeng et al., 2020). DDX3 depletion suppressed the protein expression of RAC1 in medulloblastoma (Chen et al., 2015). In the present study, we found that RAC1 expression was augmented by C1GALT1 overexpression and attenuated by C1GALT1 knockdown in LUAD cells. Rescue experiments proved that RAC1 silencing partially abolished C1GALT1-induced LUAD growth and metastasis. Hence, RAC1 was a downstream effector of C1GALT1 in LUAD. Previous studies have mainly focused on the relationship between C1GALT1 and glycoproteins (Lee et al., 2020; Kuo et al., 2021). Besides its direct function of producing aberrant glycoproteins, our current study provided new insights into the regulatory network of C1GALT1.

Our study provides a theoretical basis for C1GALT1 to be designed as a drug target and offers a novel direction for the treatment of LUAD. Indeed, there are several limitations of the current work. First, the physiological meaning and clinical application of C1GALT1 require further investigation. Second, the detailed mechanisms underlying the interaction between C1GALT1 and RAC1 need to be further investigated. Moreover, further studies are required to explore the downstream signaling pathways mediating the oncogenic effects of C1GALT1.

In summary, our study illustrated that C1GALT1 was overexpressed in LUAD tissues and facilitated the growth and metastasis of LUAD cells by upregulating RAC1. Besides, C1GALT1 expression was negatively regulated by miR-181d-5p, and decreased miR-181d-5p further contributed to C1GALT1 upregulation in LUAD. Our study highlights the importance of the miR-181d-5p/C1GALT1/RAC1 regulatory axis during LUAD progression. Accordingly, C1GALT1 may serve as a valuable prognostic biomarker for LUAD. Furthermore, targeting C1GALT1 may be an attractive therapeutic method against LUAD.

## Data Availability Statement

The original contributions presented in the study are included in the article/Supplementary Material, further inquiries can be directed to the corresponding author/s.

## Ethics Statement

The studies involving human participants were reviewed and approved by the Ethics Committee of the Hubei University of Medicine. The patients/participants provided their written informed consent to participate in this study. The animal study was reviewed and approved by the Ethics Committee of the Hubei University of Medicine.

## Author Contributions

XDo and YL designed the experiments. XDe, JS, and ST conducted the functional experiments. SC, RH, and ZL analyzed the data. CC wrote the manuscript. LS supervised the research. All authors read and approved the final manuscript.

## Conflict of Interest

The authors declare that the research was conducted in the absence of any commercial or financial relationships that could be construed as a potential conflict of interest.

## Footnotes

1 https://tcga-data.nci.nih.gov/tcga/

2 http://www.broadinstitute.org/gsea/index.jsp

## Abbreviations

NSCLC: non-small cell lung cancer
LUAD: lung adenocarcinoma
GT: glycosyltransferase
TCGA: The Cancer Genome Atlas
C1GALT1: Core 1 β1, 3-galactosyltransferase 1
RAC1: Rac Family Small GTPase 1
miR: microRNA
qPCR: quantitative Real-Time PCR
IHC: immunohistochemistry
CCK-8: Cell Counting Kit-8
3 ′ UTR: 3 ′ -untranslated region
GSEA: Gene set enrichment analysis
shRNA: short hairpin RNA
siRNA: small interfering RNA.
