# Supplementary material for: C1GALT1, Negatively Regulated by miR-181d-5p, Promotes Tumor Progression via Upregulating RAC1 in Lung Adenocarcinoma
Source: Front Cell Dev Biol. 2021 Jul 7;9:707970. doi: 10.3389/fcell.2021.707970 (PMC8292976; doi:10.3389/fcell.2021.707970)

Supplementary Material

# Supplementary Tables

# Supplementary Table 1. The sequence of shRNAs and siRNAs.

| **Name** | **Sequences (5**′**-3**′**)** |
| --- | --- |
| C1GALT1 shRNA1 | GCAAGGCATTCAGATGATAAT |
| C1GALT1 shRNA2 | GGTTGACACCCAGCCTAATGT |
| Negative control shRNA | TTCTCCGAACGTGTCACGT |
| RAC1 siRNA | GAGGCCUCAAGACAGUGUUUGACGA |
| Non-silencing siRNA | UUCUCCGAACGUGUCACGUTT |

# Supplementary Table 2. Primer sequences used for qPCR.

| **Name** |  | **Sequences (5′-3′)** |
| --- | --- | --- |
| C1GALT1 | Forward  Reverse | TAGAGGGTCCTGGTTGCTGCT  CAGTGCAGTGCTAGACATATTAC |
| RAC1 | Forward  Reverse | GGCTAAGGAGATTGGTGCTGTA  ACGAGGGGCTGAGACATTTAC |
| miR-181d-5p | Forward  Reverse | TGCGCAACATTCATTGTTGTCG  CTCAAGTGTCGTGGAGTCGGCAA |
| U6 snRNA | Forward  Reverse | CTCGCTTCGGCAGCACATATACT  ACGCTTCACGAATTTGCGTGTC |
| GADPH | Forward  Reverse | GGACCTGACCTGCCGTCTAG  GTAGCCCAGGATGCCCTTGA |

## Supplementary Figures

## Supplementary Figure 1. Survival analysis of C1GALT1 expression in the TCGA-LUSC cohort. (A) Overall survival. (B) Disease-specific survival.


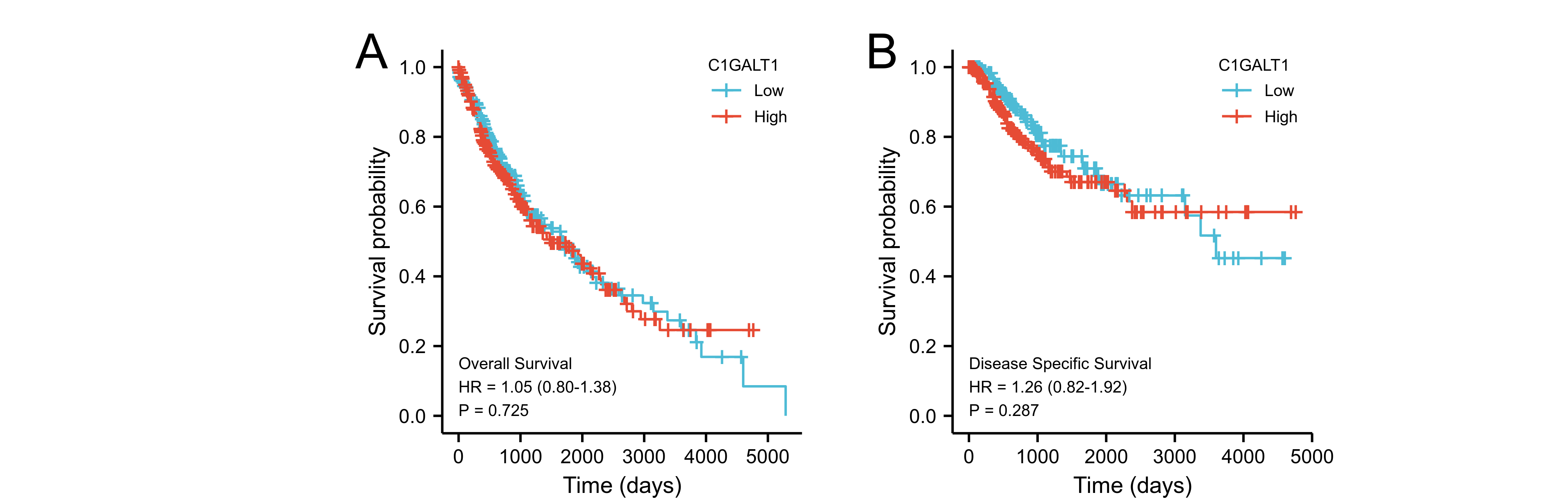


## Supplementary Figure 2. Analysis of C1GALT1 alterations in LUAD using the cBioPortal database.


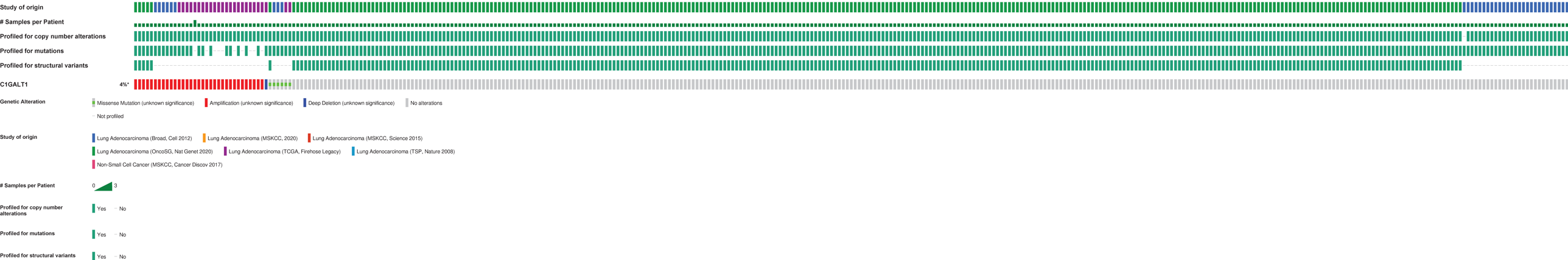


**Supplementary Figure** 3**.** The promoter methylation level of C1GALT1 in the TCGA-LUAD cohort. ^#^*p* > 0.05.


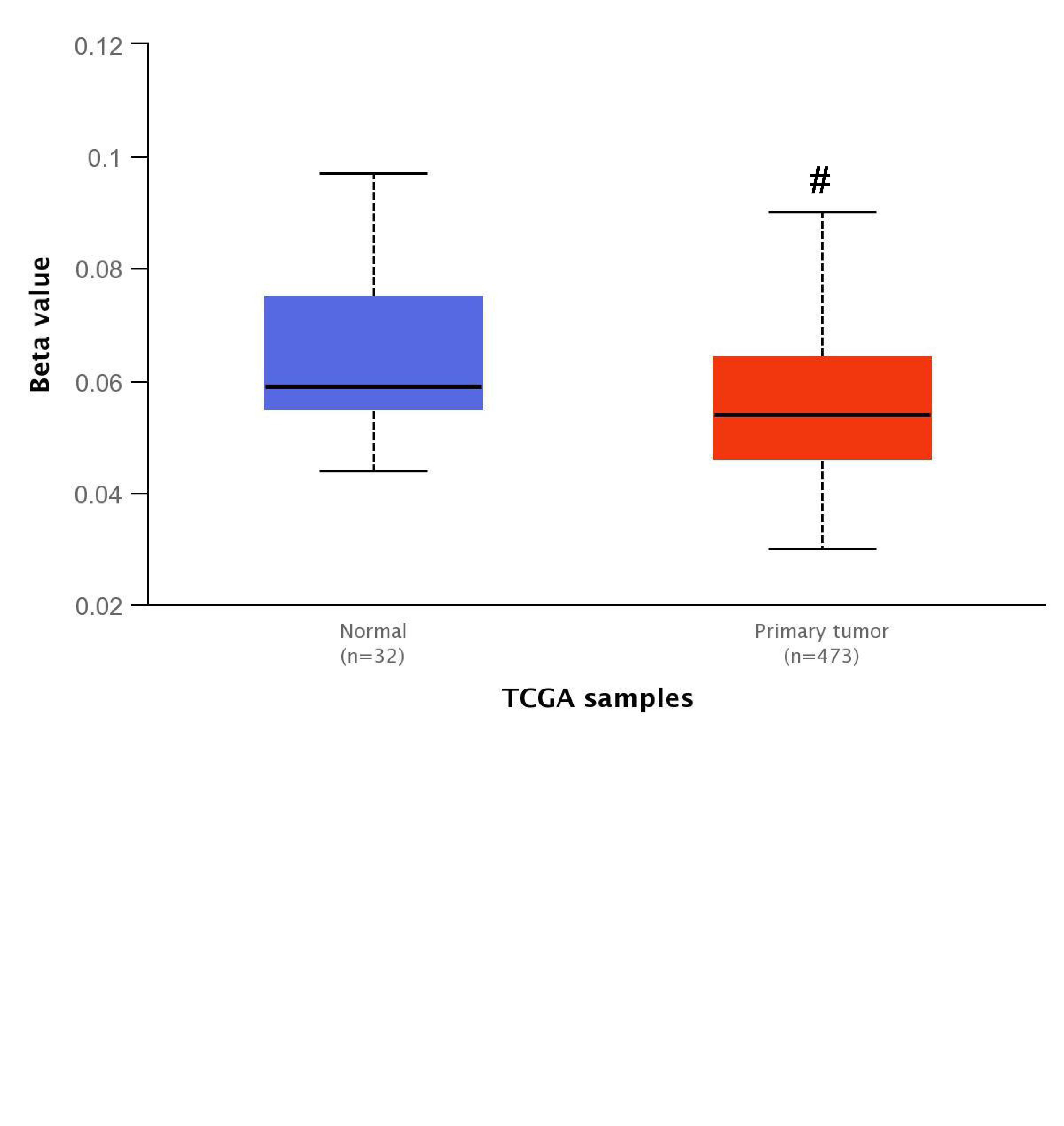


**Supplementary Figure** 4**.** TCGA database analysis of miR-148b-3p expression in LUAD. **(A)** Correlation between miR-148b-3p and C1GALT1 expression. **(B, C)** miR-148b-3p expression in unpaired **(B)** or paired **(C)** LUAD tissues and normal lung tissues. **(D)** Kaplan–Meier analysis of overall survival based on the miR-148b-3p expression. ^***^*p* < 0.001.


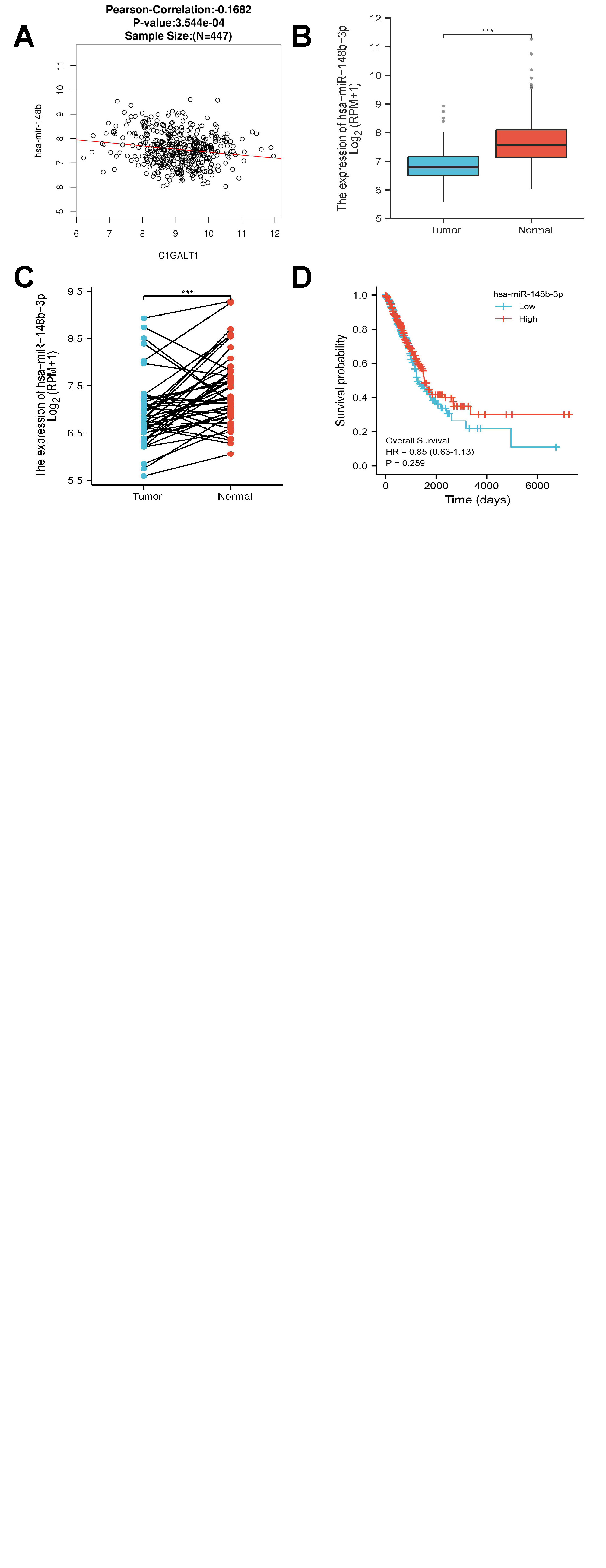


**Supplementary Figure** 5**.** Analysis of miR-181d-5p expression in LUAD cells after transfection with miR-181d-5p mimics or inhibitor by qPCR. ^*^*p* < 0.05;^**^*p* < 0.01.


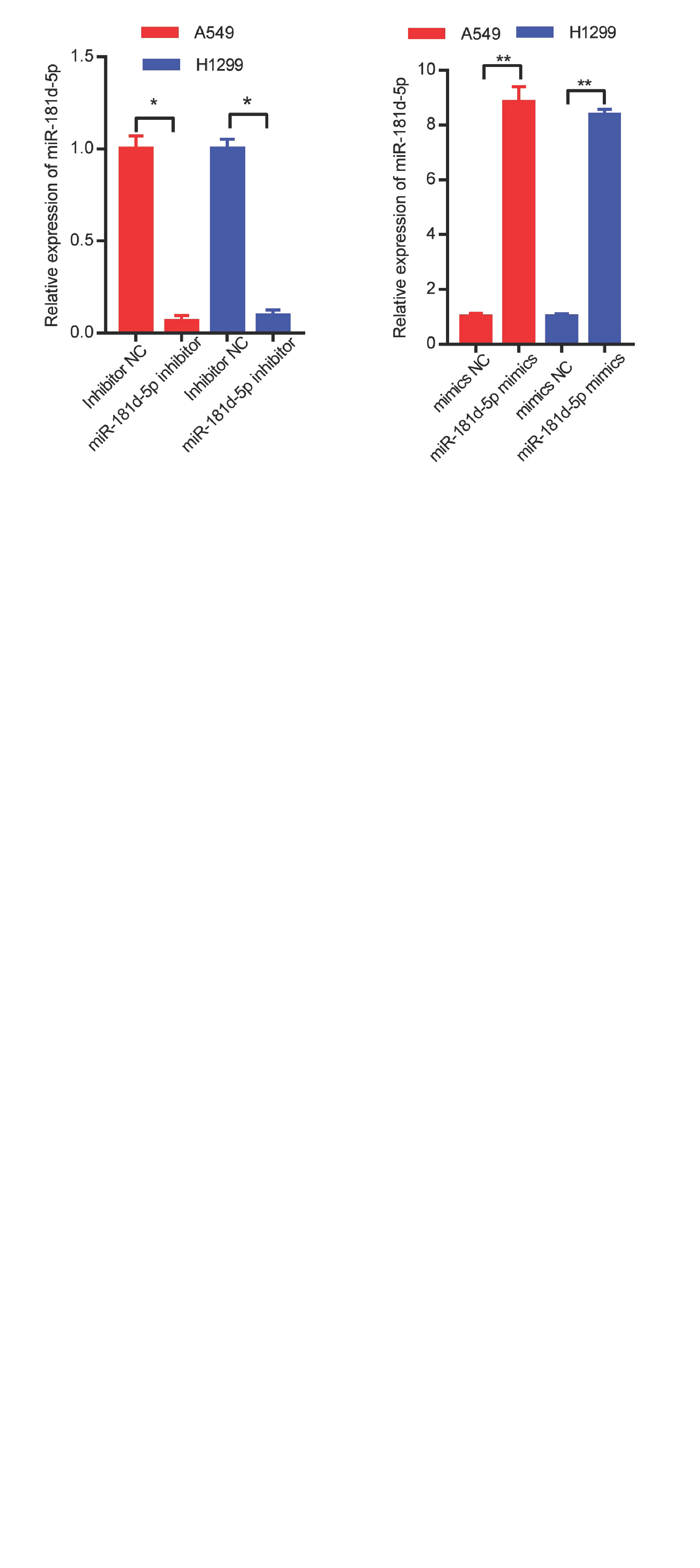


**Supplementary Figure** 6**.**Heat maps showing the top 50 genes negatively correlated with C1GALT1 in TCGA-LUAD samples.


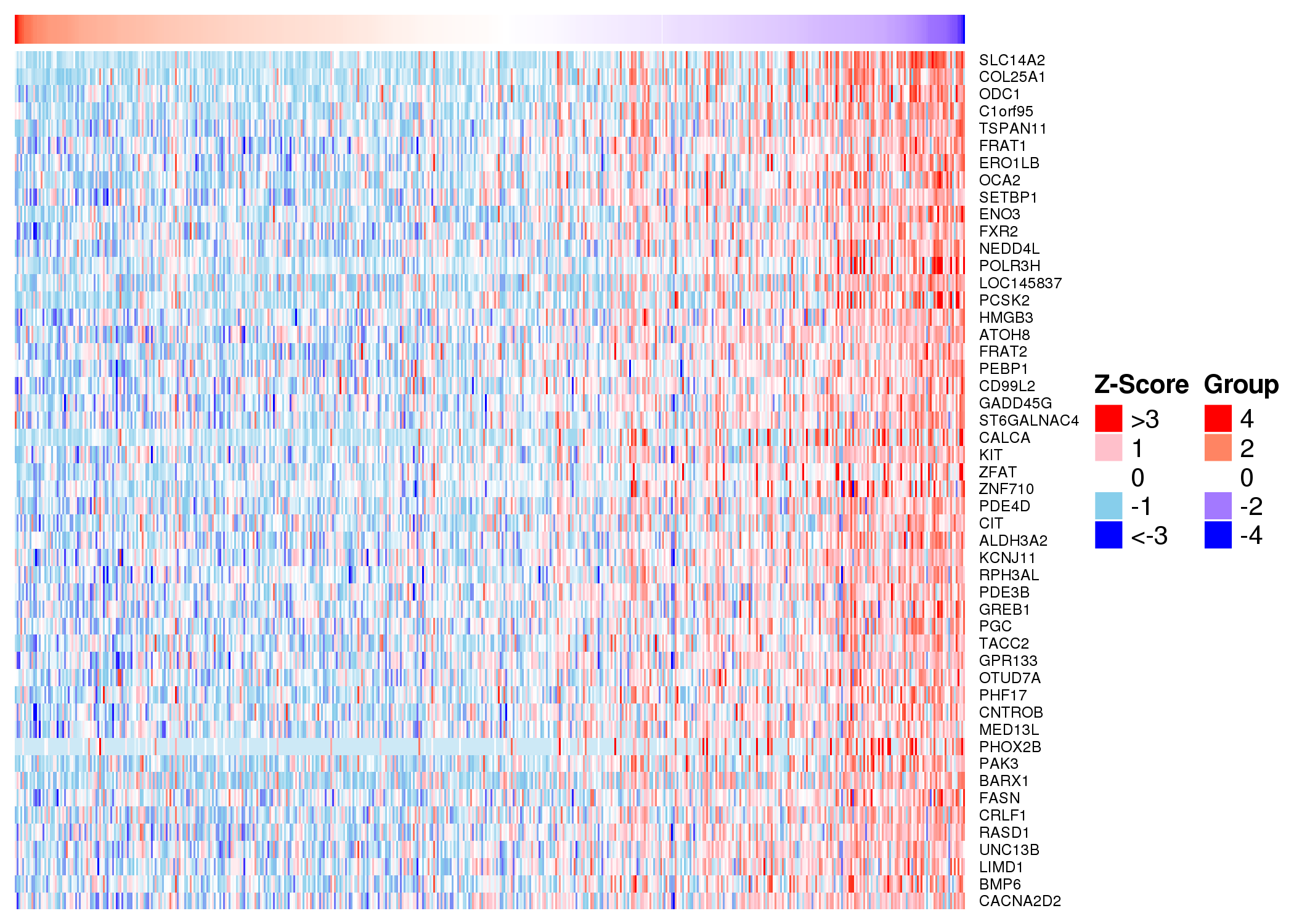


**Supplementary Figure** 7**.** The effect of C1GALT1 on RAC1 expression was detected by qPCR**.** Mock, cells transfected with empty plasmid; OV, cells transfected with C1GALT1 overexpression plasmid; shNC, cells infected with negative control shRNA; shRNA1, cells infected with C1GALT1 shRNA1. ^**^*p* < 0.01.


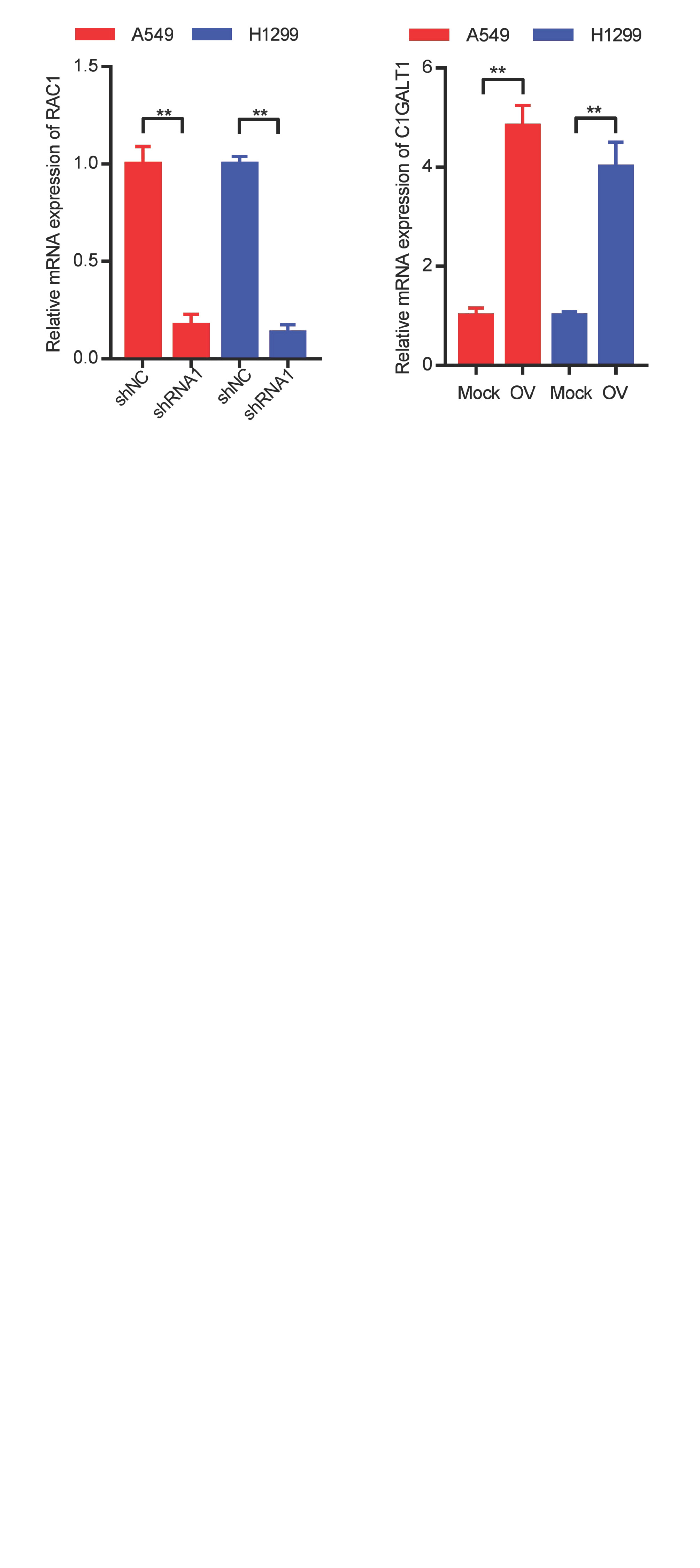

Supplement: Supplementary file 1 [file Data_Sheet_1.DOCX]
